# Oral pathogens meet the gut microbiome: new mechanistic insights on systemic disease

**DOI:** 10.3389/fcimb.2025.1673512

**Published:** 2026-02-27

**Authors:** Guowu Gan, Ruonan Chen, Peining Zheng, Kekao Long, Kenneth K. Y. Cheng, Jordy Evan Sulaiman, Xiaojing Huang

**Affiliations:** 1 Fujian Key Laboratory of Oral Diseases and Fujian Provincial Engineering Research Center of Oral Biomaterial and Stomatology Key Laboratory of Fujian College and University, School and Hospital of Stomatology, Fujian Medical University, Fuzhou, China; 2 Institute of Stomatology and Research Center of Dental and Craniofacial Implants, School and Hospital of Stomatology, Fujian Medical University, Fuzhou, China; 3 Department of Health Technology and Informatics, The Hong Kong Polytechnic University, Hong Kong, Hong Kong SAR, China

**Keywords:** gut microbiota, Porphyromonas gingivalis, Fusobacterium nucleatum, Streptococcus mutans, systemic disease, oral microbiota

## Abstract

The oral-gut axis represents a critical bidirectional pathway linking oral microbiota to systemic health. Dysbiosis of the oral microbiome, driven by pathogens like Porphyromonas gingivalis, Fusobacterium nucleatum, Streptococcus species, and Helicobacter pylori, disrupts gut ecology via direct translocation, metabolite signaling (e.g., TMAO, SCFAs), and immune crosstalk (e.g., Th17). This leads to gut barrier dysfunction, systemic inflammation, and metabolic disturbances, contributing to diverse diseases beyond the oral cavity. Evidence supports causal links with conditions including rheumatoid arthritis, cardiovascular diseases, neurodegenerative disorders, metabolic syndrome, and gastrointestinal cancers. Emerging diagnostic tools exploit these oral pathogens as biomarkers for non-invasive disease detection. Therapeutic strategies, such as probiotics, dietary interventions, and periodontal therapy, target this axis to restore microbial homeostasis and ameliorate systemic inflammation. Future research must focus on longitudinal human studies and multi-omics approaches to elucidate mechanistic details and develop effective clinical interventions for preventing and managing systemic diseases linked to oral-gut microbial dysbiosis.

## 1 Introduction

**Figure 1:**
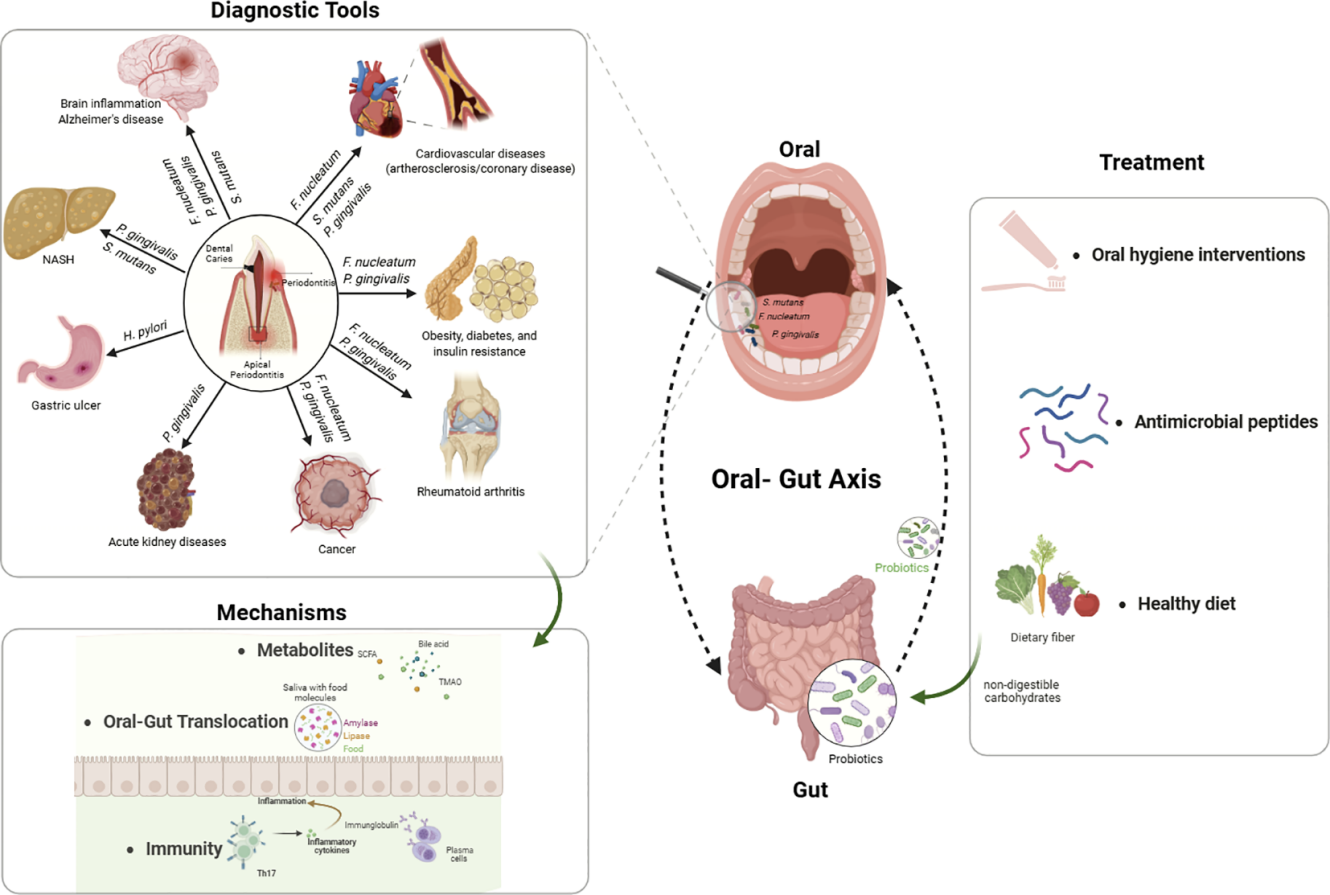
The key mechanisms of how the oral-gut axis influences systemic health. Oral pathogens (*P.g*, *F.n*, and *Streptococcus* spp.) translocate to the gut via direct ingestion or hematogenous routes, disrupting gut microbiota composition and barrier integrity. These changes trigger immune dysregulation (e.g., Th17 responses, pro-inflammatory cytokines) and metabolic disturbances (e.g., TMAO, bile acids, SCFAs), contributing to diseases such as cardiovascular disorders, Alzheimer’s disease, cancer, and metabolic syndrome. Potential interventions, including probiotics, oral hygiene, and dietary modifications, are highlighted for restoring microbial balance.

The human gut microbiota, the largest microbial community in the body, harbors over 10 trillion microorganisms. The gut microbiota is broadly classified into six major phyla: *Firmicutes*, *Bacteroidota*, *Actinobacteria*, *Proteobacteria*, *Fusobacteria*, and *Verrucomicrobia*. Dysbiosis of gut microbiota is associated with numerous systemic diseases, including rheumatoid arthritis (RA), non-alcoholic fatty liver disease (NAFLD), and diabetes (Chen and Vitetta, 2020; Lau et al., 2021; Longo et al., 2024). The human oral microbiota, the second-largest microbial community after the gut microbiota, includes 37 phyla and over 1,000 genera (Chaturvedi et al., 2025). The development of oral diseases is closely linked to ecological succession and dysbiosis of the oral microbiome. For instance, periodontal disease occurs when shifts in microbial composition lead to the overgrowth of pathogenic bacteria such as *Porphyromonas gingivalis* (*P.g*) (Śmiga and Olczak, 2025). Traditionally, the oral and gut microbiomes have been studied as separate entities. However, emerging evidence highlights the existence of a dynamic “oral-gut axis,” wherein oral microbes and their metabolites can translocate to the gastrointestinal tract, reshape gut microbial ecology, and impact host immunity and metabolism. Oral-gut dysbiosis synergistically disrupts the complement pathway, perpetuating immune evasion and inflammation (Lamont and Hajishengallis, 2015). Recent studies indicate that periodontal pathogens may contribute to gut dysbiosis and associated systemic diseases, highlighting a potential link between periodontitis and various non-oral conditions. Patients with periodontitis have been found to exhibit increased alpha diversity in both their salivary and gut microbiomes. Furthermore, alterations in the gut microbiota may impact systemic metabolite profiles, suggesting a possible mechanism by which periodontitis influences overall health (Miyauchi et al., 2025). A recent Mendelian randomization (MR) study indicated that there is a bidirectional causal relationship between gut microbiota and periodontitis (Ye et al., 2023). Despite significant progress in characterizing the oral and gut microbiota, the intricate mechanisms by which the oral-gut axis shapes systemic health remain only partially understood. Increasing evidence suggests that oral microbes and their metabolites can translocate to the gut, disrupt local microbial communities, and trigger immune and metabolic disturbances that contribute to a wide range of systemic diseases. However, the bidirectional nature of oral-gut microbial interactions, identification of robust diagnostic biomarkers, and the development of effective interventions to prevent or reverse oral dysbiosis-induced systemic effects are still major challenges in the field. This review summarizes current knowledge on the mechanistic links between the oral-gut axis and systemic health, explores the impact of key oral pathogens on gut microbiota and disease development, and discusses emerging diagnostic and therapeutic strategies of systemic diseases, as shown in **Figure 1**. By integrating insights from recent multidisciplinary research, we aim to provide a comprehensive perspective that will inform future studies and support the development of novel approaches for the prevention and management of systemic diseases driven by oral-gut microbial interactions.

## 2 Potential mechanisms by which the oral-gut axis influences systemic health

The oral and gut microbiomes are interconnected via a dynamic bidirectional relationship termed the oral-gut axis. Perturbations of this axis, such as oral dysbiosis or compromised barrier function, have been increasingly implicated in the pathogenesis of diverse systemic diseases, which involve three key mechanistic pathways of how localized oral events could exert distant effects (**Table 1**).

**Table 1:** Key mechanistic pathways linking the oral-gut axis to systemic health.

Categories	Key Mechanism	Principal Mediators/Pathways	Pathophysiological Consequences	Associated Systemic Diseases	Key References
Metabolite-Mediated Pathways	Short-Chain Fatty Acids (SCFAs)	• Acetate, Propionate, Butyrate • Oral pathobionts	• Modulate gut microbiota composition and mucosal immunity upon oral bacterial colonization. • Correlation between oral pathobiont abundance and gut SCFA levels suggests a remote signaling role.	IBD, Metabolic Disorders	(Atarashi et al., 2017; Kitamoto et al., 2020; Na et al., 2021; Wu et al., 2024; Rowland et al., 2025)
	Trimethylamine N-Oxide (TMAO)	• TMAO, Hepatic enzyme FMO3 • *P.g* LPS	• Forms an “oral-gut-liver” axis: Oral pathobionts upregulate FMO3, increasing TMAO production. • TMAO disrupts gut barrier (downregulates Claudin-1), alters microbiota, and induces systemic inflammation. • Circulating TMAO levels are dose-dependently linked to cardiovascular and liver diseases.	ASCVD, NAFLD	(Hernández-Ruiz et al., 2024; Wang et al., 2024; Budoff et al., 2025)
	Bile Acids	• Receptor signaling pathways (FXR,TGR5) • Immune cells Th17/Treg	• Affects the intestinal epithelial barrier through receptor signaling pathways (FXR/TGR5) •Promotes mucosal inflammation and barrier damage by altering intestinal epithelial cell permeability and regulating the balance of immune cells Th17/Treg.	NAFLD,NASH, Metabolic diseases	(Perino et al., 2021, Cipriani et al., 2011, Shi et al., 2023)
	Vitamin D	• Vitamin D, Vitamin D Receptor (VDR) • Antimicrobial peptides (e.g., Cathelicidin, Defensins) • Tight junction proteins (e.g., Occludin)	Oral Effects: Suppresses NF-κB, inhibits *P.g*, enhances epithelial defense. Gut & Systemic Effects: • Strengthens intestinal barrier via VDR. • Induces antimicrobial peptides and promotes anti-inflammatory Treg cells. • Deficiency is linked to oral/gut dysbiosis and cardiometabolic risks.	MetSyn, Obesity, T2DM, IBD	(Zanetta et al., 2021; Aggeletopoulou et al., 2023; Sukik et al., 2023)
Immune Crosstalk	Th17 Cell Circuit	• Th17 cells, IL-17/IL-17F cytokines • Oral pathobionts (*P.g, C. albicans*)	• Gut translocation of oral bacteria induces Th17 differentiation. Gut-derived Th17 cells can migrate back to the oral cavity. • *P.g* disrupts gut linoleic acid metabolism, promoting a pro-inflammatory Th17/Treg balance and exacerbating colitis.	Periodontitis, IBD, Oropharyngeal Candidiasis	(Nagao et al., 2022; Jia et al., 2024; Kaji et al., 2025)
	Pro-inflammatory Cytokine Storm	• IL-1β, IL-6, TNF-α • *P.g, F.n*	• Saliva from periodontitis patients upregulates colonic IL-1β and IL-6. • Oral pathogens elevate these cytokines in gut, liver, and adipose tissue, driving insulin resistance and endotoxemia. • Systemic cytokines can cross the blood-brain barrier, activating microglia and exacerbating neurodegeneration.	AD, Metabolic Disorders, IBD	(Arimatsu et al., 2014; Feng et al., 2020; Bao et al., 2022)
Direct Bacterial Translocation	Microbial Dissemination	• *P.g, Klebsiella. aeromobilis* • Periodontitis-associated microbiota	• >45% of individuals show oral bacteria in stool. ~10^8^-10¹^0^*P.g* swallowed daily can survive stomach acid and colonize the gut. • Translocated oral pathobionts directly alter gut microbiota, induce dysbiosis, and trigger local inflammation (colitis).	IBD, Gut Dysbiosis	(von Troil-Lindén et al., 1995; Segata et al., 2012; Walker et al., 2018)

### 2.1 Metabolite-mediated interactions

Research on the role of oral microbiota in metabolite-mediated interactions has progressed rapidly. These interactions can influence gut ecology and immunity through the secretion of metabolites, including short-chain fatty acids (SCFAs), trimethylamine N-oxide (TMAO), bile acids, and vitamin D. This section provides an overview of the potential mechanisms by which the oral microbiota participates in such metabolite-mediated interactions.

SCFAs are metabolites produced via fermentation of dietary fiber and resistant starch by intestinal microorganisms. They are saturated fatty acids containing 1–6 carbon atoms, including acetic acid, propionic acid, butyric acid, etc (Rowland et al., 2025). SCFAs play important roles in maintaining intestinal health, regulating immune response, and influencing host metabolism (Rowland et al., 2025). In a cross-sectional observational study, it was found that the concentration of SCFAs was correlated with the abundance of oral microbiota such as *P.g* and *Fusobacterium nucleatum* (*F.n*) (Na et al., 2021). SCFAs regulate mucosal immunity, with oral bacteria triggering SCFA-mediated immune responses upon gut colonization (Atarashi et al., 2017). SCFAs also modulate gut microbiota composition and inflammation via mucosal connections (Kitamoto et al., 2020). Systemically, *F.n* regulates intestinal butyrate metabolism through the AMPK pathway, thereby impacting the initiation, progression, and prognosis of colorectal cancer (CRC) (Wu et al., 2024).

TMAO also serves as a key metabolic mediator linking the oral microbiota, gut health, and systemic diseases. Recent findings show that the abundance of oral pathogens and plaque index are positively correlated with circulating TMAO levels (Hernández-Ruiz et al., 2024), and elevated TMAO can in turn exacerbate periodontal inflammation and alveolar bone resorption (Wang et al., 2022). Oral pathogens, especially *P.g*, can upregulate hepatic flavin-containing monooxygenase 3 (FMO3) through their derived products and promote the conversion of TMA to TMAO, while simultaneously disrupting the intestinal barrier and disturbing the gut microbiota structure, forming an “oral–gut–liver” pathogenic pathway (Wang et al., 2024; Serdar et al., 2025). At the intestinal level, TMAO can downregulate tight junction proteins, such as Claudin-1, alter the *Firmicutes/Bacteroidetes* (*F/B*) ratio, reduce the microbiota’s resilience to stress, and promote the elevation of harmful metabolites, thereby inducing systemic inflammation (Chen et al., 2022). At the systemic health level, TMAO damages the intestinal barrier and accelerates the progression of NAFLD (Nian et al., 2024). Moreover, a recent clinical study found that blood plasma TMAO levels are positively correlated with the risk of atherosclerotic cardiovascular disorder (ASCVD), and this correlation is dose-dependent (Budoff et al., 2025). This mechanistic network indicates that TMAO is not only a metabolic product of dysbiosis in the oral cavity and intestinal microbiota, but also a key molecule linking localized infection and systemic pathological states.

Accumulating evidence indicates that bile acids play a crucial role in oral-gut microbiota crosstalk and systemic disease network. In a clinical study, bile acids were detected in the gingival tissue of patients with periodontitis, and the total bile acid concentration was found to be elevated in the periodontitis group. This suggests an association between local oral events and bile acid concentration (Yang et al., 2023). At the same time, the oral pathogenic bacterium *P.g* can enter the intestine through swallowing, changing the structure of the intestinal flora, which causes an imbalance in the *F/B* ratio, thereby affecting the composition of intestinal metabolites. It further regulates genes related to bile acid synthesis in the liver, such as *Cyp7a1*, through the intestine-liver axis, ultimately exacerbating systemic metabolic disorder in diabetic mice (Kashiwagi et al., 2021). Meanwhile, studies have shown that bile acids can affect the intestinal epithelial barrier through receptor signaling pathways (FXR/TGR5) (Cipriani et al., 2011; Perino et al., 2021). Bile acid imbalance can also promote mucosal inflammation and barrier damage by altering intestinal epithelial cell permeability and regulating the balance of immune cells (Shi et al., 2023). Barrier disruption, in turn, makes bile acids and their harmful metabolites more likely to enter the systemic circulation, thereby activating metabolic and inflammation pathways in the liver and other distal organs, and promoting the progression of NAFLD/non-alcoholic steatohepatitis (NASH) and metabolic diseases, highlighting the impact of bile acids in systemic diseases (Fleishman and Kumar, 2024). Furthermore, bile acids have also been shown to regulate the interaction in the gut-brain axis (Gan et al., 2024a).Some studies have indicated an increasing trend in the abundance of *Ruminococcus* in the *P.g*-treated mouse group (Kato et al., 2018), and certain *Ruminococcus* species, such as *Ruminococcus torques* (*R.t*), possess direct vitamin D hydroxylation potential (Yamamoto and Jørgensen, 2019). To date, no studies have explicitly elucidated the relationship between the abundance of *P.g* and that of *R.t*; but this evidence-based association may provide insights into the mechanism by which oral pathogens (*P.g*) alter the composition of the intestinal flora and modulate vitamin D levels, thereby indirectly influencing the body’s vitamin D homeostasis. Vitamin D can regulate the infection and colonization processes of oral pathogens through multiple mechanisms. Existing studies have shown that vitamin D deficiency is closely associated with the loss of balance in the oral microbiota. It can maintain oral microecological homeostasis and inhibit the excessive proliferation of pathogens by suppressing the activation of the NF-κB signaling pathway and reducing the release of pro-inflammatory cytokines, such as TNF-α, IL-8, and IL-12 (Zanetta et al., 2021). In addition, vitamin D exerts a direct inhibitory effect on certain key oral pathogens. For example, it can significantly suppress the growth of *P.g* and its adhesion to host cells, while upregulating the expression of the antimicrobial peptide human β-defensin-3 (HBD-3), thereby strengthening epithelial barrier function and enhancing mucosal immune defense (De Filippis et al., 2017). These lines of evidence collectively suggest that vitamin D plays a critical role in maintaining oral microbial homeostasis. Meanwhile, an increasing number of studies have found that vitamin D also forms a bidirectional regulatory relationship with the gut microbiota through the “oral–gut axis.” On one hand, vitamin D plays a central role in maintaining intestinal barrier function. It enhances epithelial barrier integrity and prevents bacterial translocation by upregulating the expression of tight junction proteins, such as Occludin and ZO-1, via the VDR signaling pathway (Aggeletopoulou et al., 2023). At the same time, vitamin D can induce the synthesis of antimicrobial peptides, such as cathelicidin and β-defensin, enhancing the host’s ability to clear pathogenic bacteria (Cantorna et al., 2014; Aggeletopoulou et al., 2023). At the level of immune regulation, vitamin D can downregulate pro-inflammatory factors, such as TNF-α and IL-6, and promote the differentiation of regulatory T cells (Treg), thereby alleviating intestinal inflammation and indirectly stabilizing the microbial community structure (Cantorna et al., 2014; Bendix et al., 2021). On the other hand, loss of balance in the gut microbiota can lead to a reduction in bile acid synthesis, thereby affecting the uptake and metabolism of vitamin D (Li et al., 2023). This suggests that vitamin D may play a bridging role in the interaction between the oral cavity and gut microbiota. It is worth noting that microecological dysregulation associated with low vitamin D status is not limited to the oral cavity or intestinal level, but is also closely related to an increased risk of cardiometabolic diseases such as metabolic syndrome (MetSyn), obesity, and type 2 diabetes mellitus (T2DM) (Sukik et al., 2023). These findings provide new perspectives for understanding the critical role of vitamin D in the oral–gut–systemic interaction and reveal its potential as an important target for maintaining multisystem homeostasis.

### 2.2 Immune pathway crosstalk

Th17 cells, the interleukin (IL)-17 producing CD4+ T helper cells, have gained significant attention due to their crucial role as important mediators of immune dysregulation, for their production of signature cytokines including IL−17 and IL-17F under the induction by pathogens. These cytokines contribute to the recruitment and activation of other immune cells, such as neutrophils, and promote the production of additional inflammatory mediators (Zong et al., 2025). The intestinal and oral mucosa, both rich in microorganisms, have similar complex immune mechanisms to maintain a dynamic host-microbe balance, particularly Th17 immunity (Wang et al., 2023). Th17 cells may induce disease, but they also protect mucosal tissues by promoting tissue homeostasis, maintaining barrier function, and inhibiting pathogens (Wagner et al., 2021). Regulation of the IL-17/Th17 response by NLRP3, neutrophil extracellular traps, and extracellular histones leads to exacerbating bone resorption (Kim et al., 2023; Leng et al., 2023). On the other hand, Crohn’s disease (CD) patients have higher salivary Th17 cytokine levels in comparison to systemically healthy controls (Gürsoy et al., 2024). Nagao et al. discovered that gut translocation of oral pathobionts exacerbates periodontitis by enhancing Th17 cell differentiation, and oral pathobionts taken into Peyer’s patches in the gut induce peripheral Th17 responses. In turn, intestinal-derived Th17 cells migrate from the gut to the mouth upon oral infection, where the intestinal microbiome plays an important role (Nagao et al., 2022). *P.g* disrupts gut linoleic acid (LA) metabolism, activates Th17, suppresses Tregs, and exacerbates colitis, and these effects could be reversed by LA supplementation (Jia et al., 2024). Apart from *P.g*, intestinal *Candida albicans* (*C.a*) taken in by the mucosal immune system induces pathobiont-reactive Th17 cells, while these Th17 cells migrate from the gut to the mouth upon oral infection, thereby inducing Th17 responses in the tongue during oropharyngeal candidiasis (Kaji et al., 2025). However, the precise molecular mechanisms governing Th17 cell migration between the oral and gut mucosal tissues, and the specific tissue-resident signals directing their pathogenic versus protective functions, remain poorly defined.

In addition to the aforementioned IL-17, other pro-inflammatory cytokines, such as IL-1β, IL-6, and TNF-α, also play important roles in the immune interaction between the oral-gut axis and systemic diseases. Studies have shown that saliva transplantation from patients with periodontitis can induce upregulation of IL-1β and IL-6 in the colon of recipient mice, while the immunosuppressive IL-10 is downregulated, suggesting that saliva-derived microbiota may induce gut microbiota dysbiosis and low-grade intestinal inflammation through immune imbalance (Bao et al., 2022). In a previous study, oral administration of *P.g* to mice resulted in significantly increased expression of IL-1β, IL-6, and TNF-α in adipose tissue; increased expression of TNF-α and IL-6 in liver tissue; and significantly upregulated mRNA expression of IL-6 and IL-17c in intestinal tissue (Arimatsu et al., 2014). Another study also indicated that intestinal colonization by *P.g* could further elevated the expression of TNF-α in the large intestine (Nakajima et al., 2015). In addition to *P.g*, *F.n* can activate the IL-17F/NF-κB pathway via NOD2–CARD3, upregulating IL-1β, IL-6, and TNF-α, thereby exacerbating ulcerative colitis (UC) and barrier disruption (Chen et al., 2020). This suggests the role of oral microbiota in systemic diseases by disrupting the intestinal barrier. From a more macroscopic perspective, during the process of *P.g* infection and periodontitis, the above-mentioned systemic inflammatory factors are promoted to enter the central nervous system. This induces the activation of microglia and astrocytes, which in turn aggravates neurodegenerative damage and drives the progression of Alzheimer’s disease (AD) (Feng et al., 2020; Wang et al., 2023). These findings suggest that cell pro-inflammatory factors such as IL-1β, IL-6, and TNF-α are key immunological hubs through which the oral cavity microbiota affects intestinal homeostasis and drives systemic inflammation and disease progression.

### 2.3 Direct oral-gut translocation

Oral bacteria directly colonize the gut via saliva, and more than 45% of individuals in the Human Microbiome Project exhibited overlapping bacterial genera between the oral cavity and stool (Segata et al., 2012), indicating that the transfer of oral bacteria to the gut is a frequent occurrence. Oral and oropharyngeal microbes are regularly introduced into the gastrointestinal tract via swallowed saliva, food, and beverages (Segata et al., 2012). It is estimated that between 10^8^ and 10¹^0^*P.g* may be swallowed daily (von Troil-Lindén et al., 1995). If oral bacteria can withstand the acidic environment in the stomach, they can potentially survive, colonize, and multiply within the gastrointestinal tract (Walker et al., 2018). This is especially true for *P.g*, which exhibits acid resistance and may translocate to the colon, where it can alter colonic functions (Sato et al., 2017; Walker et al., 2018). Oral *F.n* translocate to the intestine by resisting the acidic environment at pH 1.5 owing to the presence of erucic acid in its cell membrane, which is regulated by FnFabM, increasing its colonization efficiency in the stomach and jejunum of mice (Li et al., 2025). Using sequencing approaches, *Parvimonas micra* was found to translocate from the subgingival sulcus of the human oral cavity to colorectal adenocarcinoma, likely migrating in a synergistic consortium with other periodontal bacteria (Conde-Pérez et al., 2024). Other periodontal pathogens, including *Klebsiella*, *Streptococcus*, and *Veillonella*, can endure harsh acidic environments and migrate to the intestine, particularly in individuals with weakened oral–gut chemical barriers (bile and gastric acid), such as infants, people with gastrointestinal disease, individuals using proton-pump inhibitors (PPIs), and elderly people (Azzolino et al., 2025; Xu et al., 2025). Similarly, Lin et al. discovered an extensive translocation of oral taxa to the rectum of populations without intestinal disorders and suggest that aging, hypertension, and PPIs use were associated with an increased abundance of oral taxa and potential pathogenic bacteria in the rectal microbiota (Lin et al., 2024). Oral bacteria can colonize the gut when swallowed in large quantities (during oral inflammation or periodontitis), with some strains surviving stomach acid through biofilm protection or inherent tolerance, while preexisting gut inflammation creates a favorable environment for their establishment by reducing microbial diversity and stability (Newman and Kamada, 2022).

The disruption of the oral microbiome may result in a higher abundance of virulent oral pathogens that can be swallowed and subsequently impact the gut microbiota and intestinal health. Periodontitis-associated salivary microbiota exacerbates colitis and anxiety in mice via gut dysbiosis and found altered metabolism of histidine in the gut and brain (Qian et al., 2023). Conversely, a metabolic labeling experiment validated that *P.g* could translocate to the gastrointestinal tract in a viable state and elicit insulin resistance, while administration of pasteurized *P.g* had no such effect (Niu et al., 2024). Germ-free mice orally administered with IBD patient saliva containing *Klebsiella aeromobilis* developed colitis by inducing the accumulation of intestinal T helper 1 cells, while the inflammatory state in IBD may render the intestine more permissive to aerotolerant oral-derived bacteria than the steady-state intestine (Atarashi et al., 2017). Therefore, direct oral-gut translocation of pathobionts via swallowed saliva can be considered to promote oral microbiota colonization in the gastrointestinal tract and disrupt gut homeostasis, thereby exacerbating intestinal inflammation and systemic manifestations. Critical questions remain regarding the *in vivo* pathogenicity threshold of translocated oral bacteria in humans, and the mechanistic interplay between oral pathobiont virulence upregulation and gut mucosal invasion. Cheng et al. utilized an *in vivo* imaging system and polymerase chain reaction (PCR) to investigate oral microbial translocation pathways. They discovered that after intra-gingival injection, fluorescently labeled *P.g* was detected in the liver, kidney, and pancreas. However, a subsequent single oral gavage experiment did not result in detectable bacteria in the blood or organs, thus proving that direct oral-gut translocation is not a pathway for systemic spread of *P.g*, although other studies using repeated oral inoculations reported *P.g* colonization in distant organs (Cheng et al., 2025). It indicates that the oral-gut axis may still be a potential route under different conditions. Future research should develop chronic exposure models that integrate host immunity, microbial ecology, and environmental factors to investigate the oral-gut axis as a dynamic pathway under real-world conditions, while employing multi-omics approaches to map pathogen translocation mechanisms and identify preventive strategies.

## 3 The effect of oral pathogens on intestinal microbiota and its role in systemic diseases

Microbial translocation studies reveal unique oral-gut axis dynamics. In 67.3% of healthy individuals, tongue-specific amplicon sequence variants (ASVs) account for 0.0%-9.37% of rectal microbiota, with *Streptococcus salivarius* (*S.s*), *F.n*, and *Streptococcus parasanguinis* (*S.p*) showing robust cross-organ colonization (Lin et al., 2024). Periodontal disease patients exhibit persistent microbial leakage from the oral cavity to the gut, though classic periodontal pathogens are not enriched, suggesting host immunity and gut microenvironment co-determine pathogenicity (Buetas et al., 2024). The effect of oral pathogens on intestinal microbiota is common even in healthy groups and could possibly be enhanced in oral disease (**Table 2**).

**Table 2:** Systemic impacts of oral pathogens via the oral-gut axis.

Oral Pathogen	Systemic Effects & Target Organs	Associated Diseases & Key Pathogenic Mechanisms	References
*Porphyromonas gingivalis*	Digestive System	Dysbiosis: Decreases microbial alpha diversity and alters beta diversity; reduces *Firmicutes/Bacteroidetes* ratio; depletes beneficial SCFA-producers (e.g., *Akkermansia*, *Lactobacillus*); enriches pro-inflammatory taxa (e.g., *Prevotellaceae*, *Proteobacteria*); induces fungal dysbiosis, enriching pathogenic species linked to altered tryptophan metabolism; downregulates tight junction proteins (ZO-1, Occludin, Claudins) and MUC2, increasing gut permeability.	(Chi et al., 2021; Tsuzuno et al., 2021; Chen et al., 2022; Dong et al., 2022; Giri et al., 2022; Park et al., 2023; Li et al., 2024; Dong et al., 2025; Oliveira-Cruz et al., 2025; Zhu et al., 2025)
		Alcoholic Liver Disease (ALD) & Non-alcoholic Fatty Liver Disease (NAFLD/MASLD): via the gut-liver axis, promoting liver inflammation, fibrosis and hepatocyte ferroptosis through LPS-TLR4 pathway and disrupted bile acid metabolism.	(Simas et al., 2021; Gao et al., 2023; Feng et al., 2025; Mousa et al., 2025)
		Inflammatory Bowel Disease (IBD): exacerbates IBD by directly degrading tight junction protein ZO-1 via gingipains, altering gut microbiota to expand IL-9-producing CD4+ T cells (driving intestinal inflammation); effects in disrupting gut homeostasis.	(Tsuzuno et al., 2021; Lee et al., 2022; Sohn et al., 2022; Huang et al., 2025a)
		Colorectal Cancer (CRC): adheres to and enriches in colonic mucosa-associated microbiota (MAM) to promote colorectal cancer (CRC) progression; accelerates CRC immune evasion by upregulating CHI3L1 in invariant Natural Killer T (iNKT) cells (impairing their cytotoxic function).	(Díaz-Basabe et al., 2024; Motosugi et al., 2025; Navarro-Sánchez et al., 2025)
		Pancreatic Cancer (PDAC): Alters the intrapancreatic microbiome, shapes a neutrophil-dominated pro-tumor microenvironment to drive PanIN-to-PDAC progression; implicated in pancreatic cancer immune escape (e.g., via miR-21 upregulation).	(Li et al., 2022a; Tan et al., 2022; Saba et al., 2024)
	Cardiovascular System	Atherosclerosis: Alters gut microbiota to upregulate bacterial carbohydrate metabolism proteins and reduce serum HDL; elevates pro-atherogenic metabolite TMAO by upregulating hepatic FMO3.	(Park et al., 2023; Wang et al., 2024)
		Aortic Plaque Formation: *P.g*-induced chronic apical periodontitis alters gut microbiota and increases TMAO, exacerbating plaques; effect transmissible via FMT.	(Gan et al., 2023; Gan et al., 2024a; Gan et al., 2024b)
	Neurological System	Alzheimer’s Disease (AD): Leads to systemic inflammation, impaired barriers, and reduced SCFAs, activating brain NLRP3 inflammasome, promoting microglial activation, and contributing to Aβ deposition and tau pathology.	(Chi et al., 2021; Qian et al., 2024; Zhang et al., 2025b)
		Parkinson’s Disease (PD): Contributes to pathophysiology by increasing systemic IL-17A and upregulating its receptor in the brain.	(Feng et al., 2020; Huang et al., 2025)
		Cognitive Impairment in T2DM: Worsens by disturbing gut microbiota and hippocampal SCFA signaling.	(Liu et al., 2025)
		Autism Spectrum Disorder (ASD): Disrupts oral and gut microbiota, leading to metabolic disturbances and exacerbation of ASD-like behaviors.	(Li et al., 2025)
	Other Systems	Metabolic Syndrome (MetSyn) & Insulin Resistance: Induces via gut dysbiosis, impaired gut barrier, and inactivated AHR signaling.	(Dong et al., 2022; Niu et al., 2024)
		Rheumatoid Arthritis (RA): Induces gut dysbiosis, disrupts intestinal barrier, promotes Th17-skewed immune response, and increases citrullinated proteins. Disruption of gut RvD5n-3 DPA-IL-10 axis is a key mechanism.	(Sato et al., 2017; Hamamoto et al., 2020; Flak et al., 2022; Kitamura et al., 2022)
		Acute Kidney Injury (AKI): Alters gut microbiota and increases gut-derived 3-indoleacrylic acid, activating neutrophils to release NGP, causing renal damage.	(Dong et al., 2025)
*Fusobacterium nucleatum*	Digestive System	Ulcerative colitis (UC): Disrupts epithelial barrier via FadA binding to E-cadherin, activating NF-κB and pro-inflammatory cytokines; triggers ferroptosis; promotes metabolic reprogramming via acetyl-CoA/STAT3.;Induces dysbiosis.	(Li et al., 2021; Li et al., 2024; Li et al., 2024; Huang et al., 2025a; Xiang et al., 2025; Zhang et al., 2025)
		Crohn’s Disease (CD): Dysbiosis.	(Connolly and Kelly, 2025)
		Colorectal Cancer (CRC): Promotes carcinogenesis by activating TLR4/NF-κB/miR-21 and PI3K-AKT-NF-κB-MMP9 pathways; induces chemoresistance; impairs antitumor immunity; promotes NET formation; targets cancer stem cells (CSCs) via Fap2 and LY6A/RPS14; initial colonization facilitated by FnEVs.	(Mima et al., 2015; Yang et al., 2017; Komiya et al., 2019; Zhang et al., 2019; Cavallucci et al., 2022; Idrissi Janati et al., 2022; Kong et al., 2023; Jia and Chen, 2024; Wang et al., 2024; Wu et al., 2024; Zheng et al., 2024; Choi et al., 2025; Wang et al., 2025)
		Pancreatic Cancer: Levels significantly elevated in fecal samples and cyst fluid from patients with IPMN; associated with oncogenesis and poor prognosis.	(Gaiser et al., 2019; Tavanaeian et al., 2025)
	Other Systems	Alzheimer’s Disease (AD): Oral infection induces gut dysbiosis, systemic inflammation, and neuropathology (increased Aβ1-42, p-Tau) via gut-brain axis.	(Yan et al., 2022; Zhu et al., 2024)
		Myocardial Injury: Co-colonization with *Lactobacillus* in diabetic mice worsens myocardial ischemia-reperfusion injury.	(Li et al., 2024)
*Streptococcus* spp.	Neurological System	Parkinson’s Disease (PD): Orally administered *S.m* exacerbates motor dysfunction and neurodegeneration in PD mice via oral/gut dysbiosis and immune activation.	(Bai et al., 2023)
	Digestive Systems	Inflammatory Bowel Disease (IBD): Significantly increased in IBD patient fecal samples; common genus linking oral and gut inflammation.	(Abdelbary et al., 2022)
		Non-alcoholic Steatohepatitis (NASH): Enriched in stools of NASH patients as an endogenous ethanol-producer.	(Mbaye et al., 2023)
	Other Systems	Type 1 Diabetes (T1DM): Reduced ‘mouth-to-gut’ transfer of *S. salivarius* linked to competition with *S.m*.	(Kunath et al., 2022)
		Chemotherapy Response: *S.m* gut abundance is a marker for responders to lung cancer chemotherapy.	(Zhao et al., 2021)
*Helicobacter pylori*	Extra-Gastric Diseases	Intestinal & Systemic: Contributes to intestinal inflammation, colorectal carcinogenesis, lung microbiome alterations, neurological disorders (post-stroke depression), and impaired pancreatic islet function in T2DM.	(Bretto et al., 2024; Deng et al., 2024; Engelsberger et al., 2024; Siegel et al., 2024; Sun et al., 2024; Wang et al., 2024; Albush et al., 2025; He et al., 2025; Li et al., 2025; Wu et al., 2025; Wu et al., 2025; Yin et al., 2025)
Other Pathogens	Intestinal System	Colorectal Cancer (CRC): Enrichment of oral pathobionts; secretes membrane vesicles triggering NF-κB-mediated inflammation, ROS, and DNA damage.	(Löwenmark et al., 2024; Miyakawa et al., 2024)
		Crohn’s Disease/*C.diff* Infection: Pro-inflammatory LPS suppresses bile acid transporter ASBT, impairing bile acid reabsorption.	(Yang et al., 2025)
	Inflammation	HIV & Oral Cancer: Increases epithelial barrier permeability, promotes microbial translocation, and drives monocyte activation.	(Fulcher et al., 2025; Nayyar et al., 2025)
	Neurological System	Migraine & PD: Oral dysbiosis features distinct taxa that form ecological clusters in the gut; periodontitis-associated oral dysbiosis occurs independently of gut changes in PD.	(Yay et al., 2024; Cho et al., 2025)
	Metabolic Disease	MAFLD & Obesity: Yellow tongue coating correlates with oral *Fusobacterium/Leptotrichia* and gut *Dialister/Eisenbergiella;* salivary microbiota provides clear obesity-related patterns.	(Bombin et al., 2022; Lu et al., 2022)

### 3.1 Porphyromonas gingivalis

The pathogenesis of *P.g* extends far beyond the confines of the oral cavity, establishing a complex “Oral-Gut-Systemic Axis” that underpins its role in a wide array of chronic diseases. This systemic impact initiates when *P.g*, a keystone periodontal pathobiont, translocates to the gastrointestinal tract via the habitual swallowing of saliva and oral biofilm, or through hematogenous spread during transient bacteremia induced by routine activities like chewing or tooth brushing (Mousa et al., 2025; Navarro-Sánchez et al., 2025). Its success as a gut colonizer is facilitated by unique virulence strategies; notably, its ability to utilize methemoglobin and bacterial hemoproteins as a heme source provides a competitive advantage over resident gut microbes (Olczak et al., 2024). This is further exemplified by its HmuY protein, which can efficiently sequester heme from *Bacteroides fragilis* proteins like BfrA, potentially disrupting microbial mutualism in the gut and exacerbating dysbiosis (Antonyuk et al., 2023). Upon reaching the gut, *P.g* exhibits a pronounced tropism for the intestinal mucosa, demonstrating a higher adhesive capacity to intestinal epithelial cells compared to other oral bacteria like *Prevotella intermedia*, an action primarily mediated by its gingipain proteases (Motosugi et al., 2025).

The ectopic colonization of *P.g* acts as a primary instigator of profound gut microbiota dysbiosis. This disruption is consistently characterized by a decrease in microbial alpha diversity and significant shifts in beta diversity, patterns observed across numerous studies and particularly exacerbated in aged hosts (Chi et al., 2021; Giri et al., 2022; Dong et al., 2025). Taxonomically, there is a frequent decrease in the Firmicutes/Bacteroidetes ratio, a reduction in beneficial SCFA-producing genera such as *Akkermansia*, *Lactobacillus*, *Clostridiaceae*, and *Muribaculaceae*, and a concomitant increase in pro-inflammatory taxa including *Prevotellaceae*, *Proteobacteria*, and pathobionts like *Mucispirillum schaedleri* (Dong et al., 2022; Park et al., 2023; Li et al., 2024). This dysbiosis is not limited to the bacterial community; *P.g* administration also induces significant fungal dysbiosis (mycobiome), enriching pathogenic species such as *Pyricularia pennisetigena*, *Alternaria alternata*, and *Candida glabrata*, which correlate with alterations in host metabolic pathways, especially tryptophan metabolism (Chen et al., 2022). A pivotal consequence of this multifaceted dysbiosis is the breakdown of the intestinal epithelial barrier. *P.g* and its metabolites directly and indirectly compromise gut integrity by downregulating the expression of tight junction proteins (ZO-1, Occludin, Claudins) and mucins (MUC2), leading to increased intestinal permeability (Tsuzuno et al., 2021; Oliveira-Cruz et al., 2025; Zhu et al., 2025).

The compromised gut barrier facilitates the translocation of microbes and their products into the systemic circulation, triggering a cascade of immunoinflammatory responses. A hallmark of *P.g*’s systemic effect is the disruption of immune homeostasis, notably a skewing towards a pro-inflammatory state characterized by an imbalance between Th17 cells and Treg cells in the gut, mesenteric lymph nodes, and spleen (Jia et al., 2024; Qu et al., 2025). Crucially, the gut microbiome is essential for priming this response, as germ-free mice do not develop *P.g*-specific Th17 cells or severe periodontitis upon challenge, a response that is restored upon microbiota recolonization (Nagao et al., 2022). This gut-primed, *P.g*-specific Th17 cell population can even migrate back to the oral cavity to exacerbate periodontal bone loss, illustrating a dynamic “gut-mouth axis” (Nagao et al., 2022). Furthermore, *P.g* ingestion disrupts critical metabolic axes within the gut. It suppresses the production of gut-protective mediators like resolvin D5n-3 DPA (RvD5n-3 DPA) and the linoleic acid (LA) metabolic pathway, which normally function as aryl hydrocarbon receptor (AHR) ligands to promote Treg differentiation and suppress Th17 responses, thereby exacerbating inflammation (Flak et al., 2022; Jia et al., 2024). Concomitantly, *P.g*-induced dysbiosis alters the metabolism of tryptophan, reducing beneficial AHR ligands and increasing metabolites like 3-indoleacrylic acid and N’-formylkynurenine, which have been linked to neutrophil activation and neurotoxicity, respectively (Niu et al., 2024; Dong et al., 2025; Zhu et al., 2025). The reduction in beneficial SCFAs such as acetate, propionate, and butyrate further impairs immune regulation and gut-brain communication (Sun et al., 2024; Liu et al., 2025).

Through these interconnected mechanisms, such as dysbiosis, barrier dysfunction, immune dysregulation, and metabolic shift, *P.g* orchestrates its impact on a spectrum of systemic diseases. In the realm of cardiometabolic diseases, *P.g* promotes atherosclerosis by altering gut microbiota to upregulate bacterial carbohydrate metabolism proteins and reduce serum HDL (Park et al., 2023). It significantly elevates the pro-atherogenic metabolite TMAO by upregulating hepatic FMO3, independent of changes in gut TMA-lyase activity (Wang et al., 2024). The role of the gut as a mediator is unequivocally demonstrated in models of chronic apical periodontitis (CAP), where *P.g*-induced CAP alters gut microbiota (increasing *Lachnospiraceae*, *Ruminococcaceae*) and increases TMAO, thereby exacerbating aortic plaque formation; an effect transmissible via FMT (Gan et al., 2023; Gan et al., 2024a; Gan et al., 2024b). *P.g* also induces metabolic syndrome (MetS) and insulin resistance by disrupting gut microbiota, impairing the gut barrier, and inactivating AHR signaling (Dong et al., 2022; Niu et al., 2024). This metabolic dysfunction extends to the liver, where *P.g* exacerbates both alcoholic liver disease (ALD) and non-alcoholic fatty liver disease (NAFLD/MASLD) via the gut-liver axis, promoting inflammation, fibrosis, and even hepatocyte ferroptosis through mechanisms involving the LPS-TLR4 pathway and disrupted bile acid metabolism (Simas et al., 2021; Gao et al., 2023; Feng et al., 2025; Mousa et al., 2025). However, there was a research finding that periodontitis driven by pathogens like *P.g* causes periodontal microbiota dysbiosis and systemic insulin resistance without major gut microbiota alterations (Blasco-Baque et al., 2017).

The neurological and neuropsychiatric systems are profoundly affected via the gut-brain axis. *P.g*-induced gut dysbiosis leads to systemic inflammation, impaired barrier function (both intestinal and blood-brain), and reduced SCFAs, which in turn activate the NLRP3 inflammasome in the brain, promote microglial activation, and contribute to amyloid-β deposition and tau pathology, exacerbating AD (Chi et al., 2021; Qian et al., 2024; Zhang et al., 2025b). Similarly, it contributes to Parkinson’s disease (PD) pathophysiology by increasing systemic IL-17A and upregulating its receptor in the brain (Feng et al., 2020; Huang et al., 2025). In the context of T2DM, *P.g* worsens cognitive impairment by disturbing the gut microbiota and hippocampal SCFA signaling (Liu et al., 2025). The impact even extends to autism spectrum disorder (ASD), where *P.g* administration disrupts oral and gut microbiota, leading to metabolic disturbances (reduced SCFAs, accumulation of 3-phenylpropionate) and exacerbation of ASD-like behaviors (Li et al., 2025). The enhanced oral-gut viral transmission observed in obese T2D individuals further underscores the breakdown of microbial barriers in systemic disease (Xu et al., 2025).

In autoimmune and inflammatory conditions, *P.g* is a major environmental trigger for rheumatoid arthritis (RA). It aggravates RA by inducing gut dysbiosis, disrupting the intestinal barrier, and promoting a Th17-skewed immune response, often accompanied by increased generation of citrullinated proteins (Sato et al., 2017; Hamamoto et al., 2020; Kitamura et al., 2022). The disruption of the gut RvD5n-3 DPA-IL-10 axis is a key mechanism by which *P.g* weakens the gut barrier and permits its arthritogenic actions (Flak et al., 2022). *P.g* also exacerbates inflammatory bowel disease (IBD) by directly degrading the tight junction protein ZO-1 via its gingipains, and by altering gut microbiota to expand IL-9-producing CD4+ T cells, which drive intestinal inflammation (Tsuzuno et al., 2021; Lee et al., 2022; Sohn et al., 2022). Its effect is potentiated in polybacterial consortia, with *P.g* exhibiting predominant pro-inflammatory effects in disrupting gut homeostasis (Huang et al., 2025a).

Perhaps most alarmingly, *P.g* plays a direct role in oncogenesis. It adheres to and enriches in the colonic mucosa-associated microbiota (MAM), promoting colorectal cancer (CRC) progression (Motosugi et al., 2025; Navarro-Sánchez et al., 2025). In CRC, *P.g* accelerates tumor immune evasion by upregulating CHI3L1 in invariant Natural Killer T (iNKT) cells, impairing their cytotoxic function (Díaz-Basabe et al., 2024). Remarkably, *P.g* translocates from the oral cavity to the pancreas, where it remains viable, alters the intrapancreatic microbiome, and promotes the progression from pancreatic intraepithelial neoplasia (PanIN) to invasive pancreatic ductal adenocarcinoma (PDAC) by shaping a neutrophil-dominated pro-tumor microenvironment (Tan et al., 2022; Saba et al., 2024). Oral-gut pathogens, including *P.g*, are broadly implicated in pancreatic cancer immune escape through mechanisms like miR-21 upregulation (Li et al., 2022a).

The systemic reach of *P.g* is further evidenced by its effects on other organ systems. It exacerbates acute kidney injury (AKI) by altering gut microbiota and increasing the gut-derived metabolite 3-indoleacrylic acid, which activates neutrophils to release neutrophilic granule protein (NGP), causing renal damage (Dong et al., 2025). During pregnancy, experimental periodontitis induces intestinal morphological changes and systemic inflammation, potentially linking oral health to adverse pregnancy outcomes (Gonzalez et al., 2025). Intriguingly, not all systemic effects are detrimental; *P.g*-induced intestinal dysbiosis can, in some contexts, elevate SCFAs and expand Tregs, thereby prolonging allogeneic skin graft survival, illustrating the context-dependent complexity of its immunomodulation (Mei et al., 2023). Finally, the interconnectedness of these systems is highlighted by bibliometric analysis, which confirms the strong research focus on the relationship between periodontitis-related bacteria like *P.g* and systemic conditions like RA (Lin et al., 2025), and by the observation that drugs like Mesalazine, used for IBD, may modulate the oral-gut axis to treat periodontitis (Wang et al., 2025b).

In conclusion, the systemic pathogenesis of *P.g* is a paradigm of modern medicine, where a localized oral infection acts as a powerful upstream driver of systemic health. Its ability to exploit the oral-gut axis, induce a multifaceted dysbiosis, disrupt critical barriers, and reprogram host immunity and metabolism, positions it as a significant contributory factor in the global burden of cardiovascular, metabolic, neurological, autoimmune, and neoplastic diseases.

### 3.2 Fusobacterium nucleatum

*F.n*, an oral commensal, influences gut and systemic health via the oral–gut axis. Under physiological conditions, oral pathogens such as *F.n* can reach the gastrointestinal tract through swallowed saliva and may colonize the gut during states of dysbiosis. Under conditions of oral dysbiosis such as periodontitis, *F.n* can disseminate to distant sites, and its enrichment in the gut is a hallmark of a disrupted microbial state, rarely observed in healthy individuals (Connolly and Kelly, 2025; Rausch et al., 2025). The translocation process is facilitated by the bacterium’s remarkable ability to withstand gastric acid, a trait conferred by erucic acid [C22:1(n9)] in its cell membrane, the synthesis of which is regulated by the *FnFabM* gene, thereby enhancing its survival and colonization efficiency in the intestine (Li et al., 2025). Furthermore, the use of PPIs significantly promotes this oral-to-gut transmission, creating a niche for *F.n* to thrive in the lower gastrointestinal tract (Zhu et al., 2024). Once the gut ecosystem is compromised, specific subspecies, particularly *F.n* subsp. *animalis* (Fna), and within it, the C2 clade, demonstrate a superior ability to colonize, driven by genetic factors like iron transporters and acid resistance mechanisms that are less prevalent in other subspecies or clades like Fna C1 (Krieger et al., 2024; Zepeda-Rivera et al., 2024; Connolly and Kelly, 2025).

Upon ectopic colonization in the gut, *F.n* acts as a potent driver of IBD. Meta-analyses confirm that oral inoculation with *F.n* significantly aggravates colitis, with this bacterium exhibiting one of the strongest detrimental effects (Akash et al., 2025). In models of UC, *F.n* exacerbates intestinal barrier dysfunction and disease severity. This is achieved through multiple, interconnected mechanisms. A primary mechanism is the disruption of the epithelial barrier via its virulence adhesin FadA, which binds to E-cadherin on host cells, leading to the activation of the NF-κB pathway and the upregulation of pro-inflammatory cytokines such as TNF-α, IL-1β, and IL-6 (Li et al., 2021; Li et al., 2024). Beyond classic inflammation, *F.n* also triggers novel cell death pathways; it activates ferroptosis, characterized by glutathione depletion, lipid peroxidation, and mitochondrial dysfunction, thereby accelerating UC pathogenesis (Zhang et al., 2025). Additionally, it promotes metabolic reprogramming in host cells, exacerbating colitis through acetyl-CoA accumulation and subsequent STAT3 acetylation and activation (Xiang et al., 2025). The resulting gut dysbiosis is characterized by a loss of beneficial microbes (*Lactobacillus*, *Bifidobacterium*) and an expansion of pro-inflammatory pathobionts (*Escherichia-Shigella*, *Enterococcus*), which further fuels the inflammatory cycle (Li et al., 2024; Huang et al., 2025a). Importantly, the detrimental impact of *F.n* on the gut community is not limited to UC but also extends to CD, where specific populations like *polymorphum* are enriched, an effect more pronounced in male hosts (Connolly and Kelly, 2025).

The pathogenic role of *F.n* is even more pronounced in CRC, where it functions as a bona fide “oncomicrobe”. Its presence is significantly elevated in CRC tissues and stool samples compared to healthy controls, and identical strains can be found in the saliva and tumors of the same patient, confirming the oral origin of gut-colonizing strains (Komiya et al., 2019; Idrissi Janati et al., 2022; Wu et al., 2024). *F.n* promotes colorectal carcinogenesis through a vast arsenal of mechanisms. It directly manipulates host signaling pathways to enhance proliferation and survival; for instance, it activates the TLR4/NF-κB/miR-21 axis to downregulate RASA1 and drive tumor growth (Yang et al., 2017), and its adhesin RadD binds to CD147 to activate the PI3K-AKT-NF-κB-MMP9 pathway (Jia and Chen, 2024). A critical aspect of its oncogenicity is its ability to induce chemoresistance. It inhibits chemotherapy-induced pyroptosis by promoting YAP nuclear translocation and BCL2 expression via the Hippo pathway (Wang et al., 2024) and confers resistance to 5-fluorouracil by upregulating BIRC3 through TLR4/NF-κB signaling (Zhang et al., 2019). Moreover, *F.n* is a master regulator of the tumor microenvironment (TME). It impairs adaptive antitumor immunity by recruiting myeloid-derived suppressor cells and reducing CD3+ T-cell density in tumor tissue (Mima et al., 2015). It also promotes neutrophil infiltration and neutrophil extracellular trap (NET) formation via TLR4-ROS signaling, which in turn enhances angiogenesis and metastasis (Kong et al., 2023). Furthermore, it disrupts mucosal immunity by impairing IgA plasma cell development, allowing for an increased intratumoral bacterial burden (Choi et al., 2025). Perhaps one of its most insidious roles is in targeting cancer stem cells (CSCs). It binds to CSCEACAM-1 via Fap2, activating NF-κB and Wnt/β-catenin signaling to enhance stemness and chemoresistance (Cavallucci et al., 2022). Recently, it was found to hijack regenerative processes by binding to the LY6A receptor on revival stem cells, upregulating RPS14, and driving their transformation into tumor-initiating cells (Wang et al., 2025). The initial colonization of CRC tissues is facilitated by extracellular vesicles derived from *F.n* (FnEVs), which transfer the outer membrane protein FomA to cancer cells, creating a niche for bacterial adhesion and aggregation (Zheng et al., 2024).

The systemic impact of oral-derived *F.n* extends far beyond the gut. In pancreatic cancer, its levels are significantly elevated in fecal samples and cyst fluid from patients with intraductal papillary mucinous neoplasms (IPMN), associating with oncogenesis and poor prognosis (Gaiser et al., 2019; Tavanaeian et al., 2025). In the context of the brain, oral infection with *F.n* in AD models induces gut dysbiosis, systemic inflammation, and neuropathology, including increased Aβ1–42 and p-Tau expression, without direct brain detection, implicating the gut-brain axis (Yan et al., 2022; Zhu et al., 2024). It also plays a role in cardiovascular complications; in diabetic coronary heart disease, increased oral *F.n* correlates with gut *Lactobacillus* and exacerbates myocardial ischemia-reperfusion injury (Li et al., 2024). Its reach even includes adverse pregnancy outcomes and atherosclerosis, often through hematogenous spread (Krieger et al., 2024; Jiang et al., 2025).

In conclusion, the translocation of *F.n* from its oral niche to the gut represents a pivotal event in systemic disease pathogenesis. It functions as a microbial driver that disrupts intestinal homeostasis, directly manipulates host cellular signaling, and reshapes the local and systemic immune landscape. The convergence of evidence across IBD, CRC, and other conditions highlights this bacterium as a critical node linking oral health to systemic pathology. This understanding opens avenues for novel strategies, such as targeting specific virulence factors, such as FadA, RadD, using precise antibiotics like metronidazole to re-sensitize tumors (Dong et al., 2021), or employing fecal microbiota transplantation to restore a healthy microbial balance and eliminate the pathobiont (Li et al., 2024). Future research focusing on subspecies-level dynamics and host-microbe metabolic crosstalk will be crucial for developing targeted interventions against this oral-origin pathobiont.

### 3.3 Streptococcus spp.

A bidirectional connection exists between the oral and gut microbiomes in patients with Parkinson’s disease (PD) (Jo et al., 2022), demonstrating the systemic pathway for oral bacteria. Notably, specific oral *Streptococci*, particularly *Streptococcus mutans* (*S.m*), exhibit a remarkable ability to translocate and survive gut transit; this species can withstand the unfavorable acidic conditions of the stomach, colonize the gut, and subsequently cause gut dysbiosis (Mukherjee et al., 2025). This gut colonization by oral *streptococci* has profound systemic consequences. Evidence shows that orally administered *S.m*, along with *Veillonella parvula* (*V.p*, enriched from human subgingival plaque), exacerbated motor dysfunction and neurodegeneration in MPTP-induced PD mice by causing oral and gut dysbiosis, plus brain and systemic immune activation (Bai et al., 2023). Furthermore, administration of specific *Streptococcus* subspecies (*S.oralis subsp. dentisani*, *S.parasanguinis*, *S.salivarius*) resulted in their gut colonization and exacerbated myocardial infarction (Li et al., 2024). Supporting the role of oral-to-gut translocation in cardiovascular disease, gut microbial species commonly found in the oral cavity were associated with detrimental plasma metabolites and coronary artery calcification. For instance, five species identified in saliva (*S.anginosus*, *S.parasanguinis*, *S.gordonii*, *R.mucilaginosa*, *B.dentium*) were positively associated with their fecal abundance and subclinical coronary atherosclerosis (Sayols-Baixeras et al., 2023). Lantibiotic bacteriocins produced by the widespread cariogenic bacterium *S.m* in the oral cavity have also been shown to perturb the intestinal microbiota, suggesting a direct mechanism by which these oral bacteria contribute to gut dysbiosis (Yonezawa et al., 2021).

Beyond cardiometabolic and neurological impacts, *Streptococcus* is consistently implicated in gut inflammation. It is the only common genus significantly increased in fecal samples from IBD patients, and genetic relatedness between oral and intestinal strains suggests oral transmission and gut colonization (Abdelbary et al., 2022). This dysbiosis extends to liver disease, where endogenous ethanol-producing bacteria, including *S.m*, are enriched in the stools of NASH patients (Mbaye et al., 2023). Alterations in the oral microbiome, such as those seen in type 1 diabetes mellitus (T1DM), impact the lower gut, exemplified by reduced ‘mouth-to-gut’ transfer of *S.s* linked to its competition with *S.m*, potentially contributing to inflammatory processes (Kunath et al., 2022). Significant gut microbiota compositional differences overlapping with oral health markers, like severe dental caries and sarcopenia, further underscore this systemic link (Yang et al., 2023). In addition, responses to medical interventions are influenced, as *S.m* abundance in the gut microbiome was identified as a bacterial marker relevant to responders to lung cancer chemotherapy (Zhao et al., 2021). However, a study showed a small leakage of some oral bacteria, mainly *streptococci* like *Streptococcus salivarius*, to the gut, regardless of periodontal health status, but the proportion of oral bacteria in feces was very low, which suggested that periodontitis alone may not be sufficient to promote significant translocation and colonization of oral pathogens in the gut; instead, other host factors like aging, water intake, antibiotic use or preexisting gut inflammation must be present (Buetas et al., 2024).

Collectively, this evidence strongly implicates the translocation and colonization of oral *Streptococcus* species within the gut as a key driver of dysbiosis contributing to diverse systemic pathologies.

### 3.4 Helicobacter pylori

*Helicobacter pylori* (*H.p*) infection exerts its impact on the microecology starting from the oral cavity, which serves as a significant reservoir in sites such as dental plaque, carious lesions, and root canals, potentially acting as a source for gastric infection and transmission (Hirsch et al., 2012; Iwai et al., 2019; Almashhadany et al., 2022; Abdul et al., 2023). This infection not only significantly disturbs the oral microbiota balance but also exacerbates caries development by altering biofilm structure. Specifically, *H.p* can co-localize with and utilize the biofilm formed by *S.m*, creating an environment favorable for the proliferation of cariogenic bacteria (Zhang et al., 2018; Nomura et al., 2020). Clinical studies further confirm a moderate to strong association between oral *H.p* infection and higher caries severity, specifically manifested as higher DMFT/DMFS scores, ICDAS II code 6 lesions, and an increased number of affected teeth (Aksit Bıcak et al., 2017; Hamada et al., 2019; Iwai et al., 2022; Sruthi et al., 2023), accompanied by poorer periodontal indicators (Urban et al., 2017). Notably, *H.p* can transiently survive in saliva, and its survival ability is significantly enhanced in the presence of *S.m* and *Actinomyces naeslundii* (Scholz et al., 2025), providing a possibility for transmission from the oral cavity to the stomach.

This oral microbiota imbalance can further compromise the gastric environment and promote the colonization of oral bacteria such as *F.n* and *P.g* in the human gut (Xu et al., 2025). A substantial body of evidence demonstrates that *H.p* infection induces significant disruptions to the gut microbiota, characterized by an enrichment of pro-inflammatory bacteria such as *Clostridioides difficile, Proteobacteria, Fusobacterium* spp., and *Prevotella* spp., alongside a depletion of beneficial taxa including *Lactobacillus* spp.*, F.prausnitzii*, and *Bacteroides* (Albush et al., 2025; Li et al., 2025; Wu et al., 2025; Yin et al., 2025). This dysbiosis is critically linked to *H.p*-related disease progression, serving as both a marker for severity and a potential therapeutic target (Wu et al., 2025).

The impact of these microbial alterations extends far beyond the stomach, participating in the pathogenesis of various extra-gastric conditions through the disruption of immune homeostasis and induction of gut microbiota alterations. These conditions include intestinal inflammation and colorectal carcinogenesis (Engelsberger et al., 2024; Wu et al., 2025). Furthermore, the influence of *H.p* reaches distant organs via axes such as the gut-lung axis, where infection in infancy can alter the lung microbiome and associated inflammation (Siegel et al., 2024), and the microbiota-gut-brain axis, thereby increasing the risk of neurological disorders such as post-stroke depression (Sun et al., 2024; Wang et al., 2024). *H.p*-induced gut microbiota alterations have also been implicated in impairing pancreatic islet function in T2DM patients (He et al., 2025).

Interestingly, the role of *H.p* in immune-mediated diseases exhibits a complex and dualistic nature; while epidemiological evidence often suggests a protective role against autoimmune diseases like IBD by promoting immune tolerance, the concomitant microbial dysbiosis, particularly the increase in bacteria like *Proteobacteria* and *Actinobacteria*, may conversely exacerbate intestinal inflammation (Bretto et al., 2024; Deng et al., 2024). Consequently, optimizing treatment to prevent refractory infection and exploring microbiota-modulating strategies are highlighted as crucial steps in management (Li et al., 2025).

Apart from dental caries and periodontitis, *H.p* infection is also linked to oral diseases such as oral lichen planus and aphthous stomatitis. It alters the diversity and stability of the oral microbiota and interacts with key oral pathogens. *H.p* cooperates with *S.m* to promote caries, enhances the virulence and inflammatory response of *P.g* in periodontitis, and survives within *C.a*, which provides it with shelter, helping it resists environmental stresses and antibiotics, thereby aiding its persistence and reinfection (Fan et al., 2025). The clinical implications of this oral reservoir are profound. The presence of oral *H.p*, particularly in untreated dental caries, has been associated with failed gastric eradication therapy and can negatively impact systemic health, including a synergistic effect with periodontitis on accelerating memory decline in older adults following an inflammatory diet (Iwai et al., 2024; Chou et al., 2025). Therefore, maintaining good oral hygiene and receiving professional periodontal and caries treatment are considered crucial for eradicating and preventing the recurrence of gastric *H.p* infections (Almashhadany et al., 2022; Cuba et al., 2025). However, it is important to note that some studies have not found a significant association between *H.p* in dental plaque and the stomach in the same individual, or between the bacterium and caries incidence in certain populations, highlighting the complexity of this relationship (Mehdipour et al., 2022; Moosavian et al., 2023).

### 3.5 Other pathogens

Beyond the well-established roles of *P.g, F.n*, *Streptococcus* spp., and *Helicobacter pylori*emerging evidence implicates other oral microorganisms in modulating the oral-gut axis, with profound systemic implications.

For instance, 16S rRNA sequencing of fecal samples revealed a distinct microbial shift in CRC patients compared to controls, characterized by a higher abundance of oral pathobionts including *Parvimonas micra, Peptostreptococcus stomatis*, and various *Porphyromonas* species (Löwenmark et al., 2024). The study reveals that oral bacterial networks, including pathogens like *Fusobacterium* and *Peptostreptococcus*, colonize the colonic mucosa in CRC and CD, forming similar co-abundance structures at both sites. This colonization is negatively associated with *Lachnospiraceae* abundance, suggesting its protective role against pathogenic oral taxa and possibly mediated through habitual diet (Flemer et al., 2018). *Actinomyces odontolyticus*, an oral bacterium abundant in the gut of early colorectal cancer patients, secretes membrane vesicles that trigger NF-κB-mediated inflammation and mitochondrial dysfunction, resulting in excessive ROS production and DNA damage. This process promotes cellular transformation and contributes to colorectal carcinogenesis (Miyakawa et al., 2024). The oral commensal *Veillonella* emerges as a critical pathobiont. In CD, its pro-inflammatory LPS suppresses the bile acid transporter ASBT via TLR4 and MAPK–c-Jun/c-Fos signaling, impairing bile acid reabsorption and exacerbating *Clostridioides difficile* infection (Yang et al., 2025). Similarly, *Veillonella parvula* is enriched in both oral cancer and HIV, where it increases epithelial barrier permeability, promotes microbial translocation, and drives monocyte activation and systemic inflammation (Fulcher et al., 2025). This genus is also more abundant in IBD patients, alongside *Streptococcus*, suggesting its role as a microbial marker for gut inflammation (DeClercq et al., 2025). In terms of oral cancer, oral saliva microbes such as *Haemophilus parainfluenzae, Veillonella parvula*, and *Rothia mucilaginosa* were strongly associated with malignancy, while in stool samples, certain *Faecalibacterium* spp were associated with the benign group, suggesting a species-dependent manner of association, whereas *Ruminococcus* and *Eubacterium* spp. were linked to potentially malignant and malignant groups (Nayyar et al., 2025). These microbial shifts indicate that oral and gut microbiomes could act as potential biomarkers, aiding in early detection and assessment of oral cancer risk.

Conversely, the gut microbiome itself influences oral health. MR analyses reveal causal relationships, with specific gut taxa (*Eubacterium brachy* group and *Terrisporobacter*) increasing dental caries risk, while others (*Escherichia-Shigella* and *Oscillibacter*) are protective (Wang et al., 2025a). Moreover, gut microbial alterations, such as reduced *Akkermansia muciniphila* in severe periodontitis, can be restored via periodontal therapy or FMT, improving gut barrier function and ameliorating periodontal inflammation (Zhang et al., 2025). FMT from healthy donors also alleviates diabetic periodontitis by reducing alveolar bone loss and oxidative stress (Gong et al., 2025).

The transmission of oral microbes to the gut further underscores this axis. Maternal periodontitis expands oral pathobionts like *Klebsiella aerogenes*, which translocate to the infant gut, disrupt intestinal immunity, and predispose offspring to enteritis (Haraguchi et al., 2025). Similarly, oral dysbiosis in migraine patients features increased *Gemella* and *Rothia* and decreased *Veillonella*, with these oral taxa forming distinct ecological clusters in the gut, implying oral-gut migration (Cho et al., 2025).

In systemic diseases, oral-gut microbial interactions are prominent. In metabolic-associated fatty liver disease (MAFLD), yellow tongue coating correlates with oral *Fusobacterium* and *Leptotrichia*, which interact with gut *Dialister* and *Eisenbergiella*, reflecting carbohydrate metabolism disorders and inflammation (Lu et al., 2022). Obesity similarly reshapes oral and gut microbiomes, with salivary microbiota providing clearer obesity-related patterns than fecal microbiota, highlighting oral sampling’s utility (Bombin et al., 2022). Furthermore, polybacterial periodontal infections, such as the ‘red complex’, impair the BH4/nNOS/NRF2 pathway, reducing nitric oxide bioavailability and disrupting colonic motility, thereby linking oral pathogens to gastrointestinal dysfunction (Gangula et al., 2015). In respiratory diseases, Yan et al. demonstrated that oral-originated gut *Prevotella* would influence childhood asthma through immune activation via molecular mimicry and modulation of host lipid metabolism (Yan et al., 2024).

Oral-gut microbial crosstalk also extends to neurodegenerative conditions. Molar extraction in mice disrupts gut microbiota and promotes neuroinflammation via L-Asparagine (Ji et al., 2025), while adolescents with caries and obesity exhibit oral-gut-brain axis imbalances that drive psychological disorders (Wang et al., 2025). Dysbiotic oral microbiota characterized by significantly reduced Firmicutes and Bacteroides levels alongside increased Actinomycetes in children with cerebral palsy and epilepsy (CPE) can translocate to the intestinal tract, inducing gut microbiota dysbiosis where *Bifidobacterium, Bacteroides*, and *Prevotella* emerge as dominant genera (Huang et al., 2022), Notably, in PD, periodontitis-associated oral dysbiosis occurs independently of gut microbial changes, underscoring disease-specific oral effects (Yay et al., 2024). Oral, not gut microbiota diversity, also reflects the inflammation and neoplasia in patients with uveitis and vitreoretinal lymphoma (Brichova et al., 2025). Oral microbiota dysbiosis in depression, characterized by increased *Pseudomonas* and decreased *Streptococcus*, directly alters gut microbiota composition (elevated *Muribaculaceae* and reduced *Lachnospiraceae*), leading to systemic effects such as barrier dysfunction and depression-like behaviors through the microbiota-oral-brain axis (Lou et al., 2024).

The oral-gut axis thus serves as a critical mediator in systemic inflammation and disease progression, with specific microbial taxa offering potential biomarkers for early detection, as seen in oral cancer where *Haemophilus parainfluenzae* and *Rothia mucilaginosa* are malignancy-associated (Nayyar et al., 2025). Overall, targeting these microbial interactions may yield novel interventions for systemic diseases.

## 4 Future directions

### 4.1 Diagnostic tools for systemic diseases

In recent years, an increasing number of studies have revealed that oral bacteria and their specific molecular products are closely associated with various systemic diseases through oral-gut axis. By detecting the composition, abundance, and specific gene or protein expression levels of oral microbiota, it is possible to reflect an individual’s health status and disease risk. For example, changes in the abundance of *P.g*, *F.n*, and *Streptococcus* spp., as well as their specific molecular markers, have been shown to correlate with the onset, progression, and severity of multiple diseases. Compared with traditional imaging or blood biochemical tests, oral microbiome analysis is not only non-invasive and easily repeatable, but also reflects microecological changes and underlying mechanisms of disease, offering new perspectives for personalized disease prevention and treatment. Specifically, the salivary microbiota of patients with colorectal polyps showed an increased abundance of harmful bacteria including *P.g, F.n, Leptotrichia wadei, Prevotella intermedia, and Megasphaera micronuciformis*, while beneficial bacteria such as *Prevotella nanceiensis* decreased (Zhang et al., 2023). Combined with machine learning, gut microbiome signatures, such as enrichment of such CRC-associated pathogens, can potentially act as a scalable and non-invasive approach for early CRC and adenomas detection (Tsai et al., 2025). As *P.g* serves as a crucial biomarker for CRC screening and diagnosis due to its presence in malignant tissues and feces of patients, yet existing detection methods are hindered by high costs and complexity (Mei et al., 2023). Therefore, Zhang et al. developed a novel aptasensor utilizing SELEX-selected aptamers and MoS2 nanoflowers integrated with CRISPR/Cas12a amplification, which achieves a detection limit of 10 CFU/mL and validates elevated *P.g* levels in CRC patient feces, developing a highly specific and sensitive aptasensor for CRC screening and diagnosis (Zhang et al., 2025a).

Simultaneously, specific microbial biomarkers are being identified for systemic diseases linked to the oral cavity. *F.n*, rich in the saliva and colonic tissues of patients with CRC compared to healthy controls, is implicated in disease progression and poor prognosis, serving as a potential diagnostic biomarker detectable by methods including qPCR (Cheng et al., 2020; Li et al., 2022b; Negrut et al., 2023; Wang and Fang, 2023). The number of its virulence factor FadA gene is significantly elevated in CRC tissues versus healthy tissues and correlates positively with malignancy grade (Rubinstein et al., 2013; Li et al., 2022b). Salivary exosome proteomics further reveals diagnostic potential, identifying eight unique proteins in active IBD patients absent in healthy individuals (Zheng et al., 2017). Overall, as the gateway and source of microbial down-transmission to the gastrointestinal tract, the oral cavity is in a vital position (Wang et al., 2020). These bacteria and their specific molecular products are associated with systemic diseases through various mechanisms, providing new directions for the development of non-invasive diagnostic tools. However, when considering the use of oral bacteria as biomarkers to predict digestive system diseases for non-invasive diagnostic techniques, collection time and site should be taken into consideration (Wang et al., 2020).

### 4.2 Treatment of microbiota dysbiosis and systemic diseases

A growing body of evidence highlights the significant therapeutic potential of various interventions targeting the oral-gut axis to mitigate the local and systemic consequences of periodontitis. These strategies range from established clinical procedures to novel probiotics, natural compounds, and dietary modifications, all converging on the restoration of microbial homeostasis and the reduction of inflammation.

The cornerstone clinical intervention, nonsurgical periodontal treatment (NSPT), demonstrates profound systemic benefits beyond oral health. In Apoe–/– mice with *P.g*-induced periodontitis, NSPT not only alleviated alveolar bone resorption but also reduced atherosclerotic plaque area. This cardioprotective effect was mechanistically linked to a significant reduction in plasma levels of the atherogenic metabolite TMAO, achieved through the downregulation of its key synthesizing enzyme, hepatic FMO3. Furthermore, NSPT ameliorated systemic inflammation by decreasing pro-inflammatory cytokines (IL-6, TNF-α, IL-1β) and enhancing intestinal barrier integrity via upregulation of tight junction proteins (ZO-1, Occludin). The treatment also reshaped the gut microbiota, with correlation analyses indicating that specific bacterial genera associated with TMAO levels were also linked to inflammatory factors and barrier function, underscoring NSPT’s role in modulating the oral-gut axis to improve systemic health (Huang et al., 2025b).

Complementing mechanical therapy, probiotic interventions offer a promising microbial-centric approach. The probiotic *Streptococcus cristatus* CA119 exhibited dual functionality by competitively excluding periodontal pathogens like *P.g* and *F.n* from the oral cavity and protecting against their induced intestinal inflammation. This protection, observed whether CA119 was administered via oral inoculation or direct gavage, was accompanied by enhanced systemic antioxidant capacity (increased T-SOD, T-AOC; decreased MDA, H_2_O_2_, MPO) and reduced inflammatory cytokines (IL-1β, IL-18), highlighting its systemic anti-inflammatory potential (Zhao et al., 2024). This is consistent with broader findings that specific probiotic strains, such as *Lactobacillus acidophilus* LA5 and *Lacticaseibacillus rhamnosus* GG, can attenuate periodontitis-induced bone resorption and enhance bone regeneration by modulating the gut microbiota and circulating metabolites (Cataruci et al., 2024; Huang et al., 2024). Notably, the gut commensal *Akkermansia muciniphila*, which is often reduced in periodontitis, demonstrates potent anti-inflammatory effects. Oral administration of *Akkermansia muciniphila* reduces *P.g- and F.n*-induced periodontal bone loss and inflammation by modulating host immune responses (increasing IL-10 and decreasing IL-12) and strengthening mucosal barriers in both the gingiva and intestine (Huck et al., 2020; Mulhall et al., 2022; Song et al., 2023). Crucially, this protective effect is dependent on oral contact and is amplified through gut microbiome remodeling, highlighting a local-systemic interplay via the oral-gut axis. In a specific systemic context, supplementation with *Bifidobacterium animalis subsp. lactis* HN019 in pregnant mice attenuated periodontitis-induced intestinal damage, systemic inflammation, and adverse pregnancy outcomes like fetal growth restriction (Gonzalez et al., 2025), while *Clostridium butyricum* MIYAIRI 588 alleviates periodontal bone loss in diabetic mice via gut microbiota regulation (Zhang et al., 2023a). The therapeutic scope of probiotics extends to metabolic health, with oral supplementation of *Bifidobacterium* breve improving glycemic control in T2DM and *Anaerobutyricum soehngenii* enhancing intestinal barrier function through SCFA production, improving metabolic indicators in prediabetes (Shetty et al., 2020; Chaiyasut et al., 2023; Attaye et al., 2025). A synthetic microbial community (SynCom) designed by metabolic network reconstruction promotes *F.n* decolonization, and enhances tryptophan metabolism and secondary bile acid conversion, leading to reduced lipid accumulation, decreased inflammatory reaction, and enhanced tumor inhibition effect, providing a promising approach for treating *F.n*-positive CRC (Zhou et al., 2025). Zhang et al. also developed recombinant *Lactobacillus plantarum* that expresses FomA protein, a critical pathogenic element of *F.n*, demonstrating its potential in protecting mice from severe IBD induced by *F.n* through the oral-gut axis by its oral administration to stimulate protective immune responses in the gut (Zhang et al., 2023). Beyond whole bacteria, gut-derived metabolites represent another innovative approach. For example, KetoC, a metabolite produced by *Lactobacillus plantarum* in the gut, exerts direct antimicrobial effects against *P.g*. Daily oral administration of KetoC attenuates alveolar bone loss by disrupting the bacterial membrane integrity of periodontopathogens, providing a precise mechanism to manage oral dysbiosis without the collateral damage of broad-spectrum antibiotics (Sulijaya et al., 2019).

Natural compounds and botanicals present another viable intervention strategy. Epimedium polysaccharide alleviated *P.g*-aggravated gut dysbiosis and intestinal inflammation by directly inhibiting the pathogen’s growth and virulence, restoring beneficial gut genera (*Akkermansia, Bifidobacterium*), and suppressing the pro-inflammatory Th17 pathway (Li et al., 2024). Similarly, paeoniflorin mitigated periodontitis-associated systemic inflammation by restoring the Th17/Treg balance, suppressing the IL-6/STAT3/IL-17 pathway, and reversing gut microbial dysbiosis. Its efficacy was further linked to the modulation of key metabolic pathways, including arginine biosynthesis and arachidonic acid metabolism, which are correlated with inflammation (Qu et al., 2025). Inonotus hispidus polypeptide also demonstrated efficacy by simultaneously rebalancing both the oral and gut microbiota, increasing health-associated genera (oral *Rothia* and gut *Oscillospira*) while decreasing pathogenic ones, thereby relieving inflammation via the oral-gut axis (Wu et al., 2025). Banxia Xiexin Decoction can inhibit *F.n* colonization by interfering with the binding of FadA to E-cadherin, reducing the activation of the E-cadherin/β-catenin signaling pathway, and ultimately delaying colitis-to-cancer transition (Jiang et al., 2024).

Notably, dietary composition emerges as a powerful modulator of the oral-gut-systemic axis. A palmitic acid-enriched Western diet (PA-ED) was shown to exacerbate *P.g*-induced gut microbiota destabilization, impair stress resilience, and enhance osteoclastic bone resorption. In stark contrast, an oleic acid-enriched Mediterranean diet (OA-ED) stabilized the gut microbiome, maintained its modularity, increased levels of the stress-resolving lipokine PI(18:1/18:1), attenuated inflammation, and limited systemic bone loss (Döding et al., 2025). This underscores the role of the gut microbiome as an immune-modulatory hub and positions nutritional intervention as a foundational strategy to mitigate the systemic impact of periodontal infection. Supporting this, intake of dietary fibers boosts the abundance of butyrate-producing bacteria, which are often depleted in periodontitis, and SCFA supplementation has been proposed to alleviate neurological conditions like AD by modulating neuronal energy metabolism and reducing neuroinflammation (Fu et al., 2019; Liu et al., 2019; Sun et al., 2023). The use of probiotics like *Lactobacillus* and *Bifidobacterium* to improve gut microbiota and ameliorate cognitive deficits in AD models further expands the systemic promise of these interventions (Hashioka et al., 2021; Abdelhamid et al., 2022; Pan et al., 2022; Zhang et al., 2023b). Beyond these, advanced interventions like nisin, an antimicrobial peptide produced by *Lactococcus lactis*, correct dysbiosis across oral, gut, and liver sites to prevent NAFLD steatosis (Kuraji et al., 2024).

Recently, ultra-small gold nanoclusters (AuNCs) proved to be a promising antibiofilm agent, by targeting oral biofilms, and indirectly normalizing gut microbiota through the oral-gut axis (Zhang et al., 2022). In conclusion, the arsenal of interventions against periodontitis and its systemic effects is diverse and multifaceted. From professional periodontal debridement and specific probiotic strains to natural bioactive compounds and strategic dietary choices, these approaches collectively target the oral-gut axis to restore microbial eubiosis, reinforce barrier integrity, and quell systemic inflammation, thereby offering a holistic framework for combating the wide-ranging impacts of periodontal disease.

### 4.3 Elucidating inter-species interactions between the oral and gut microbiota

So far, there are limited studies on the relationship between oral microbes and the gut microbiota, as researchers typically consider the two as separate systems. However, with recent evidence on the importance of the oral-gut axis, it is clear that studying the interactions between oral pathogens and the gut microbiome is crucial to better understand their causal relationship and to develop prevention strategies for systemic diseases. For instance, oral infection with *P.g* exacerbates acute kidney injury by disrupting gut microbiota composition, leading to synergistic interactions among specific taxa such as *Porphyromonas*, *Staphylococcus*, and *Lachnospiraceae*, which collectively contribute to disease progression through metabolic and immune pathways (Dong et al., 2025).

#### 4.3.1 Network-based correlation analysis

Top-down network analyses have been used to statistically identify keystone taxa and disentangle microbial co-occurrence patterns in a diverse range of ecosystems, including in the human microbiome (Fisher and Mehta, 2014). This technique can be used to disentangle the relationship between the oral and gut microbiota. In correlation network-based analyses, interactions among members in communities were identified based on correlated molecular behaviors derived from metagenome-wide “omics” studies (Bruggeman and Westerhoff, 2007). This relies on various multivariate statistical analyses that have been increasingly used in the field of microbial ecology (Raes and Bork, 2008). Network-based analyses have been used to examine the mechanisms that shape the structure and function of complex real-world communities in a hypothesis-driven manner. In particular, co-occurrence networks can show the presence or absence of members in these communities, highlighting the extent to which particular taxa, metabolites, and other microbe-associated traits interact antagonistically or synergistically. In pregnant women, periodontitis induces synergistic changes in their microbial profiles via the oral-gut axis, with positive correlations between oral pathogens and specific gut bacteria such as *Coprococcus*, alongside competitive interactions with other gut genera like *Lachnoclostridium*, collectively reshaping the maternal microbial ecosystem network (Cheng et al., 2024). Shotgun metagenomic sequencing or 16S rRNA sequencing is used to determine the relative abundance of microbial taxa, and these abundance values are used to construct co-occurrence networks based on correlation metrics (Bahram et al., 2018). The correlation values can be viewed as the interaction type between two microbial taxa, with negative correlation values indicating antagonistic interactions (nutrient competition or production of growth-inhibiting metabolites) and positive values indicating synergistic interactions (mutualism or commensalism of metabolites) (Freilich et al., 2011). For instance, the butyrate-producing *Butyribacter* showed positive correlations with neuroactive metabolites L-Asparagine, while these metabolites exhibited inverse correlations with expression of neuroinflammatory genes Tbx and Arhgdib, demonstrating how tooth loss-induced depletion of *Butyribacter* led to reduced levels of neuroprotective metabolites (particularly L-Asparagine), which subsequently resulted in upregulation of neuroinflammatory markers (Tbx1 and Arhgdib), thereby establishing a mechanistic pathway from tooth loss to neuroinflammation through the oral-gut-brain axis (Ji et al., 2025). This could reveal gut species with positive and negative interactions with the oral microbes, including pathogens.

Even though these correlation values cannot directly inform the underlying mechanisms shaping the community structure or confirm the existence of interactions among microbes, the main strength of co-occurrence networks is the ability to summarize a vast array of pairwise associations into network elements (comprised of edges and nodes) and features (such as degree, closeness or betweenness centrality) to generate testable hypotheses. The network components and features can be used to identify biologically meaningful patterns and important hubs or keystone taxa within the community. Overall, network analyses of communities comprising oral and gut microbes are useful for identifying key and important members of the communities and characterizing changes in network patterns within and across conditions (e.g. disease states). However, although this method could shed new biological insights into systems that are still poorly characterized, this approach is phenomenological rather than theoretical and hence has a limited predictive capability.

### 4.3.2 Bottom-up construction of synthetic communities

Unlike top-down network-based correlation analysis, bottom-up construction of synthetic communities could be used to systematically map inter-species interactions and gain a deeper understanding of molecular and ecological mechanisms governing the interactions (Venturelli et al., 2018). In this approach, different community combinations are assembled to obtain experimental measurements (species abundance, metabolites production), which were then fitted to mechanistic or empirical models (Qian et al., 2021) to generate insights into the community interactions and function (e.g. inter-species interaction parameters) (Clark et al., 2021). Ecological models such as the generalized Lotka-Volterra (gLV) or the MacArthur consumer resource models were typically utilized to infer the interaction parameter values, but machine learning models, such as the recurrent neural networks (RNNs), have recently gained interest due to their greater flexibility compared to modeling dynamical systems (Baranwal et al., 2022; Thompson et al., 2023). Previous studies have elucidated the inter-species interactions between a human gut pathogen (e.g. *C.difficile*) and the gut commensals (Hromada et al., 2021; Sulaiman et al., 2024; Sulaiman et al., 2025), and the knowledge was then used to design anti-pathogen microbial consortia for therapeutic applications. Similar principles could be applied to studying the interactions between oral pathogens and the gut microbiota and designing defined communities that can effectively inhibit the oral pathogens.

Omics approaches could then be applied to obtain mechanistic insights into the inferred interactions. For instance, exo-metabolomics of conditioned media from monocultures and co-cultures could reveal metabolite utilization and production profiles of these microbes, hence inferring the extent of resource competition and cross-feeding (Venturelli et al., 2018; Sulaiman et al., 2025). In the tooth loss model, serum metabolomics identified L-Asparagine as a key gut-derived metabolite that accumulated systemically and thereby further substantiating the mechanistic link along the oral–gut–brain axis *in vitro* study (Ji et al., 2025). Metabolomic analysis also demonstrated that periodontitis significantly alters the fecal metabolic profile of first-trimester pregnant women, revealing potential mechanisms through which maternal periodontitis impacts the distal gut by influencing bile acid and tryptophan metabolism pathways (Cheng et al., 2024). Notably, metabolomics has been instrumental in uncovering the role of microbial metabolites in host-pathogen interactions. In childhood asthma, untargeted ultrahigh-performance liquid chromatography-mass spectrometry (UPLC-MS) metabolomic analysis of fecal samples revealed significant disruptions in lipid metabolism that were correlated with the enrichment of oral-originated *Prevotella* and *Bacteroides* species (Yan et al., 2024). Furthermore, *in vitro* treatment of human bronchial epithelial cells with *P.bivia* supernatant altered lipid profiles, promoting pro-inflammatory metabolites and suppressing anti-inflammatory lipids, thereby elucidating how microbial metabolites drive inflammatory responses in asthma (Yan et al., 2024). Additionally, untargeted metabolomics has been applied to assess serum metabolite changes induced by microbial interactions. For example, *Lactiplantibacillus plantarum* precolonization led to the upregulation of 12-oxo phytodienoic acid and downregulation of antioxidants and lipid peroxidation products, indicating reduced oxidative stress and a shift in host metabolic state that may underlie its protective effects against caries (Wu et al., 2025). Metabolomics not only identifies differential metabolites but also links them to transcriptomic changes and cellular outcomes, offering a systems-level view of microbial synergy and host response (Dong et al., 2025). Transcriptomics and proteomics in co-culture vs monoculture could reveal differentially expressed genes and proteins in the presence of the partner species compared to growth in isolation, revealing altered biological processes due to the partner species (Sulaiman et al., 2024; Kamrad et al., 2025). These would yield insights into the type of interactions that can effectively inhibit the pathogens for therapeutic purposes.

## 5 Conclusion

Collectively, based on the comprehensive evidence reviewed, this work establishes the bidirectional oral-gut axis as a fundamental mediator of systemic health. Key oral pathobionts, including *P.g*, *F.n*, *Streptococci spp*, and *H.p*, translocate to the gastrointestinal tract where they disrupt microbial ecology, compromise barrier function, and trigger dysregulated immune responses. MR analyses confirm a causal relationship between gut dysbiosis and periodontitis, highlighting their interconnected pathogenesis. These disruptions initiate inflammatory cascades contributing to diverse systemic pathologies across multiple organ systems, such as cardiovascular disease, neurodegenerative disorders, metabolic syndrome, and gastrointestinal cancers. Critical knowledge gaps remain regarding precise mechanisms governing microbial migration, host-specific susceptibility thresholds, and pathogenicity markers. Future research must prioritize longitudinal human studies to validate causal links and identify robust diagnostic biomarkers detectable in oral samples. Therapeutic strategies should target microbial translocation and immune dysregulation through microbiome-modulating interventions, engineered probiotics, and precision antimicrobials. Ultimately, understanding the oral-gut axis offers transformative potential for preventing and managing complex chronic diseases driven by this systemic crosstalk.
